# Cytotoxic T lymphocytes that recognize decameric peptide sequences of retinoblastoma binding protein 1 (RBP-1) associated with human breast cancer

**DOI:** 10.1038/sj.bjc.6690698

**Published:** 1999-09

**Authors:** T Takahashi, J Cao, D S B Hoon, R F Irie

**Affiliations:** Departments of Biotechnology Sciences Molecular Oncology, John Wayne Cancer Institute, 2200 Santa Monica Blvd, Santa Monica, CA, 90404, USA

**Keywords:** CTL, RBP-1, Rb protein, HLA, breast cancer, peptide

## Abstract

Retinoblastoma binding protein 1 (RBP-1) is a 143-kDa nuclear phosphoprotein that promotes cell growth by inhibiting the product of retinoblastoma tumour suppressor gene (pRB). We recently found that RBP-1 contains KASIFLK, a heptameric peptide (250–256) recognized by human antibodies and overexpressed by breast cancer cells. In the present study, we demonstrate that human T-cells stimulated with RBP-1 decameric peptides containing KASIFLK can kill human breast cancer cells. These decamers, GLQKASIFLK (247–256) and KASIFLKTRV (250–259), have anchor motifs for both HLA-A2 and HLA-A3. Peripheral blood lymphocytes from 41 normal donors were stimulated by these peptides in culture media containing 15 IU ml^−1^ interleukin-2, 25 IU ml^−1^ interleukin-7 and 500 IU ml^−1^ granulocyte–macrophage colony-stimulating factor. Cytotoxic activity of the T-cells was assessed against autologous B lymphoblastoid cells pulsed with each peptide. Stimulation by GLQKASIFLK generated specific cytotoxic T lymphocyte (CTL) lines from HLA-A2, A3 donors, HLA-A2 donors and HLA-A3 donors. Stimulation with KASIFLKTRV generated specific CTL lines from HLA-A2 donors. No HLA-A2^−^, A3^−^ CTL line showed specific cytotoxicity against these target cells. These CTL lines were also cytotoxic against HLA-A2 and HLA-A3 breast cancer cells but not against normal fibroblastoid cell lines, normal epidermal cell lines, or a melanoma cell line. RBP-1 peptide antigens may be of clinical significance as a potential peptide vaccine against human breast cancer. © 1999 Cancer Research Campaign


*British Journal of Cancer* (1999) **81**(2), 342–349
© 1999 Cancer Research Campaign
Article no. bjoc.1999.0698

### Cytotoxic T lymphocytes that recognize decameric peptide sequences of retinoblastoma binding protein 1 (RBP-1) associated with human breast cancer

**T Takahashi[1], J Cao[1], DSB Hoon[2] and RF Irie[1]**

Departments of [1]Biotechnology Sciences and [2]Molecular Oncology, John Wayne Cancer Institute, 2200 Santa Monica Blvd, Santa Monica, CA 90404, USA

**Summary** Retinoblastoma binding protein 1 (RBP-1) is a 143-kDa nuclear phosphoprotein that promotes cell growth by inhibiting the product of retinoblastoma tumour suppressor gene (pRB). We recently found that RBP-1 contains KASIFLK, a heptameric peptide (250–256) recognized by human antibodies and overexpressed by breast cancer cells. In the present study, we demonstrate that human T-cells stimulated with RBP-1 decameric peptides containing KASIFLK can kill human breast cancer cells. These decamers, GLQKASIFLK (247–256) and KASIFLKTRV (250–259), have anchor motifs for both HLA-A2 and HLA-A3. Peripheral blood lymphocytes from 41 normal donors were stimulated by these peptides in culture media containing 15 IU $ml^{-1}$ interleukin-2, 25 IU $ml^{-1}$ interleukin-7 and 500 IU $ml^{-1}$ granulocyte–macrophage colony-stimulating factor. Cytotoxic activity of the T-cells was assessed against autologous B lymphoblastoid cells pulsed with each peptide. Stimulation by GLQKASIFLK generated specific cytotoxic T lymphocyte (CTL) lines from HLA-A2, A3 donors, HLA-A2 donors and HLA-A3 donors. Stimulation with KASIFLKTRV generated specific CTL lines from HLA-A2 donors. No HLA-$A2^-$, $A3^-$ CTL line showed specific cytotoxicity against these target cells. These CTL lines were also cytotoxic against HLA-A2 and HLA-A3 breast cancer cells but not against normal fibroblastoid cell lines, normal epidermal cell lines, or a melanoma cell line. RBP-1 peptide antigens may be of clinical significance as a potential peptide vaccine against human breast cancer.

© 1999 Cancer Research Campaign

**Keywords:** CTL; RBP-1; Rb protein; HLA; breast cancer; peptide

Retinoblastoma (Rb) gene is a tumour suppressor gene that encodes the Rb family of proteins, which have an important role in regulating cell cycle progression (Lee et al, 1987, 1988; Bookstein et al, 1990; Wiman, 1993). Rb proteins have multiple phosphorylation sites as well as motif sequences for binding specific regulatory proteins. Phosphorylation of Rb proteins favours cell cycle progression and cell proliferation (Wiman, 1993). During proliferation, Rb protein phosphorylation increases in the late G1 phase of the cell cycle, remains high in S and G2 phases, and then dephosphorylates during mitosis. Rb proteins have broad transcriptional effects such as inhibiting E2F factor required for expression of genes involved in DNA replication (Heskcth, 1997). Mutation or deletion of the Rb gene can result in uncontrolled cell proliferation (Lee et al, 1988; Bookstein et al, 1990; Heskcth, 1997). Rb proteins interact with a variety of proteins, some of which have been well characterized: E2Fq, ATF-2, p34CDC2, insulin, lamins, PU.1 and retinoblastoma binding protein (RBP) (Defeo Jones et al, 1991; Huang et al, 1991; Kaelin et al, 1991, 1992; Heskcth, 1997).

Recently, a human IgG antibody that reacts with breast cancer cells was found to be specific to the heptameric peptide sequence KASIFLK within RBP-1 (Cao et al, 1999). Breast cancer patients had high titres of anti-RBP-1 IgG antibodies. Although the RBP family comprises RBP-1 and RBP-2, only the former expresses KASIFLK (Fattaey et al, 1993; Otterson et al, 1993). To our knowledge, this peptide sequence is not present in any other human protein sequence. The functions of RBP-1 and RBP-2 remain unknown (Fattaey et al, 1993; Otterson et al, 1993; Heskcth, 1997).

Peptides recognized by antibodies are usually shorter than eight amino acids, whereas peptides recognized by HLA class I restricted T-cells are 9–12 amino acids in length. Previously, we showed that peptide sequences recognized by human monoclonal antibodies can also be recognized by human T-cells and that the sequences are immunogenic in humans (Morioka et al, 1994; Takahashi et al, 1997*a*, 1997*b*). In the present study we hypothesized that a peptide sequence recognized by an antibody also might be recognized by cytotoxic T lymphocytes (CTL) if adjacent amino acids with HLA class I motifs were added to the antibody-recognized sequence. To test this hypothesis we synthesized decameric and nonameric RBP-1 peptides that contained KASIFLK plus adjacent amino acids with known HLA-A2 and/or HLA-A3 anchor motif residues. Three such peptides – GLQKASIFLK (247–256), KASIFLKTRV (250–259) and KASIFLKTR (250–258) – were used to stimulate peripheral blood mononuclear cells (PBMC) from human donors with HLA-A2 and/or HLA-A3 phenotype. CTL lines established by stimulation with RBP-1a and RBP-1b showed strong cytotoxic activity against HLA-A2 and/or HLA-A3 matched peptide-pulsed B lymphoblastoid cells (BLC) and against HLA-A matched established breast cancer lines. The antigenic peptide sequence expressed by RBP-1 may have potential clinical utility as a breast cancer vaccine.

*Received 22 September 1998*
*Revised 2 February 1999*
*Accepted 25 March 1999*

*Correspondence to:* RF Irie

### MATERIALS AND METHODS

### Synthetic peptides

Two decapeptides and one nonapeptide of RBP-1 were synthesized with a free COOH and $NH_2$ terminal at Research Genetics, Inc. (Huntsville, AL, USA). KASIFLKTRV and KASIFLKTR were easily dissolved in standard culture medium; GLQKASIFLK was insoluble in standard medium and therefore was dissolved in 100% dimethyl sulphoxide (DMSO) at 100 mg $ml^{-1}$ and stored at –20°C. Prior to use, all three peptides were diluted to 100 μg $ml^{-1}$ in a culture medium and stored at 4°C for no more than 2 weeks.

### Cell line establishment

Eleven breast cancer primary cultures were established from metastases of individual patients as follows: surgical specimens were minced and treated with RPMI-1640 containing 0.02% trypsin, 200 U $ml^{-1}$ of DNAase I and 0.15 g $ml^{-1}$ of collagenase (Sigma, St Louis, MO, USA) for 2 h at 37°C to make a single-cell suspension. Cells were seeded to 12-well culture plates and cultured in RPMI-1640 supplemented with 10% heat-inactivated fetal calf serum (FCS) (Gemini Bioproducts, Calabasas, CA, USA) plus penicillin G, streptomycin and amphotericin B (Gibco-BRL, Grand Island, NY, USA). Non-adherent cells were removed 2 days later. A homogeneous cancer cell population was selectively developed by cloning and passaging. These cells were maintained in the above medium supplemented with insulin and transferrin. JWCI-BA, a long-term (> 30 passages) breast cancer cell line that expressed the breast cancer-associated KASIFLK antigen was HLA-A2, A3, which was maintained in the same culture medium was used in certain experiments. Fibroblastoid and epidermoid primary cultures representative of normal cell controls were established as previously described. Epidermal cells were obtained by scraping a thin layer of epidermis, which was separated from dermis by culturing overnight with RPMI-1640 containing 0.3% trypsin. JWCI-MA, which is a KASIFLK-negative, HLA-A2, A3 melanoma cell line (> 20 passages), was also used as a control. All cell lines were subjected to immunohistochemical analysis using a human anti-KASIFLK IgG antibody. All control cell lines were proven to be negative for the KASIFLK antigen, and all breast cancer cell lines were positive for the antigen. Viral-transformed BLC lines were established from normal donors by incubating PBMC with Epstein–Barr virus (EBV) obtained from supernatant of B95-8 marmoset BLC line (Morioka et al, 1994). HLA typing of all cell lines was carried out by HLA antibody microcytotoxicity assay (Dr Paul Terasaki's laboratory at the UCLA School of Medicine) and/or by DNA polymerase chain reaction (PCR) assay. These assays tested peripheral blood lymphocytes from donors of the cell lines.

### Generation of CTL lines

PBMC were obtained from fresh blood of 41 normal healthy donors by fractionation with Ficoll Hypaque gradient (Pharmacia Biotech, Alameda, CA, USA). PBMC ($3 \times 10^6$ per well) were cultured in a 24-well culture plate with AIM-V media (Gibco- BRL) containing 10% human AB heat-inactivated serum (Gemini Bioproducts) and 10 μg $ml^{-1}$ of synthetic peptide. After 2 days, half of the culture medium was replaced with fresh medium supplemented with 15 IU $ml^{-1}$ (final concentration) of interleukin-2 (IL-2) (R&D Systems, Minneapolis, MN, USA) and 25 IU $ml^{-1}$ of interleukin-7 (IL-7) (R&D Systems). PBMC were transferred to new wells to remove adherent cells. This procedure was repeated every 2 days to completely remove adherent cells before the next peptide stimulation. After 6 days, $3 \times 10^6$ of cryopreserved autologous PBMC were thawed and incubated for 6 h with culture medium containing 500 IU $ml^{-1}$ of granulocyte–macrophage colony-stimulating factor (GM-CSF) (R&D Systems) and 10 μg $ml^{-1}$ of the peptide. PBMC were then irradiated at 5000 rads and non-adherent cells were removed and used as feeder cells. Responder cells were added to these adherent cells and then cultured. Two days later, half of the medium was replaced with fresh medium containing IL-2 and IL-7, and the responder cells were transferred to new wells. The same procedure was repeated. At day 30, all responder cells were tested for cytotoxicity against autologous EBV-transformed BLC pulsed with peptide. Successful peptide-specific CTL lines were maintained for more than 16 weeks by restimulation with peptide and cytokines every 6th day.

### $^{51}$Cr-release cytotoxic assay

$^{51}$Cr-release cytotoxic assay was performed as previously described (Morioka et al, 1994). Target cells were labelled with 100 μCi of $Na_2{}^{51}CrO_4$ (Amersham, Arlington Heights, IL, USA). BLC targets were prepulsed with 10 μg $ml^{-1}$ of the peptide for 2 h at 37°C before effector cells were added. The percentage of specific lysis was calculated as follows: 100 × [(experimental release−spontaneous release)/(maximum release−spontaneous release)]. Spontaneous release was determined by incubating target cells with medium only, and maximum release was determined with 3% Triton X-100. Spontaneous release and maximum release were counted in BLC targets with and without peptide; no differences were observed. All values were the average from triplicate samples.

### Monoclonal antibody blocking

An antibody blocking assay was performed to assess CTL specificity. $^{51}$Cr-labelled target cells were incubated at 37°C with IgG murine monoclonal antibody (mAb) against HLA class 1, HLA-DR, and HLA-DQ (AMAC, Inc., Westbrook, ME, USA); HLA-A2 (clone BB7.2 ATCC); or HLA-A3 (clone GAP A3 ATCC). After 30 min, effector cells were added, and $^{51}$Cr-release CTL assay was performed as described above.

### Cold target inhibition assays

Effector cells were incubated with unlabelled cold targets (fibroblasts, PBMC, K562 cells and JWCI-BA breast cancer cell line) at specific ratios in 96-well round-bottom microtitre plates for 1 h at 37°C. $^{51}$Cr-labelled hot targets were then added to the wells and incubated at 37°C for 4 h. The E:T ratio was constant at 40:1.

### RESULTS

### HLA-A2 or HLA-A3 restricted recognition of RBP-1 peptide by CTL

The peptide sequences selected in this study – GLQKASIFLK, KASIFLKTRV, and KASIFLKTR – were derived from the natural RBP-1 protein sequences 247–256, 250–259 and 250–258, respectively, and contain HLA-A2 and/or HLA-A3 motifs adjacent to the

© 1999 Cancer Research Campaign

**Table 1** HLA-restricted recognition of RBP-1 peptides by CTL derived from 12 normal donors

| PBMC donors[a] | HLA-A type | Autologous BLC pulsed with: | | | | | |
|---|---|---|---|---|---|---|---|
| | | GLQKASIFLK[b] | | KASIFLKTRV | | KASIFLKTR | |
| | | + | – | + | – | + | – |
| 1 | 1, 1 | 3.7 ± 0.3[c] | 3.3 ± 0.3 | 1.4 ± 0.1 | 0.9 ± 0.1 | 3.5 ± 0.3 | 3.8 ± 0.3 |
| 2 | 1, 2 | **42.7 ± 1.8** | 2.9 ± 0.3 | **26.6 ± 1.4** | 4.1 ± 0.4 | 0.0 ± 0.1 | 0.0 ± 0.2 |
| 3 | 1, 3 | **30.8 ± 1.4** | 3.1 ± 0.3 | 6.7 ± 0.5 | 7.2 ± 0.4 | 2.2 ± 0.2 | 2.1 ± 0.1 |
| 4 | 1, 24 | 5.4 ± 0.2 | 6.0 ± 0.4 | 3.5 ± 0.3 | 3.2 ± 0.3 | 4.4 ± 0.4 | 4.6 ± 0.3 |
| 5 | 2, 2 | **48.5 ± 1.9** | 5.1 ± 0.3 | **29.4 ± 0.9** | 8.9 ± 0.6 | 0.6 ± 0.2 | 1.5 ± 0.1 |
| 6 | 2, 3 | **58.9 ± 1.8** | 3.8 ± 0.3 | **35.1 ± 1.4** | 3.8 ± 0.2 | 5.3 ± 0.5 | 4.6 ± 0.3 |
| 7 | 2, 32 | **53.6 ± 21.5** | 7.2 ± 0.5 | **17.6 ± 0.9** | 2.4 ± 0.2 | 3.9 ± 0.3 | 3.3 ± 0.3 |
| 8 | 3, 11 | **29.3 ± 1.0** | 1.8 ± 0.2 | **13.7 ± 0.9** | 2.5 ± 0.2 | **11.8 ± 0.7** | 2.4 ± 0.3 |
| 9 | 3, 31 | **37.4 ± 1.0** | 6.5 ± 0.5 | 3.2 ± 0.3 | 3.6 ± 0.3 | 1.4 ± 0.2 | 2.0 ± 0.3 |
| 10 | 3, 68 | **24.2 ± 1.4** | 4.7 ± 0.2 | 0.8 ± 0.1 | 0.3 ± 0.1 | 4.2 ± 0.3 | 2.6 ± 0.2 |
| 11 | 25, 33 | 1.1 ± 0.2 | 1.9 ± 0.2 | 4.2 ± 0.4 | 3.8 ± 0.2 | 3.1 ± 0.3 | 2.5 ± 0.2 |
| 12 | 29, 30 | 4.3 ± 0.3 | 3.5 ± 0.3 | 2.9 ± 0.2 | 3.4 ± 0.3 | 1.7 ± 0.1 | 1.1 ± 0.2 |

[a]PBMC from normal donors were stimulated for 4 weeks with each of the three RBP-1 peptides and tested for their cytotoxicity using $^{51}$Cr-release assay. [b]Target cells were autologous EBV-transformed BCL with and without each peptide. [c]Data are expressed as average per cent specific cytotoxicity and standard deviation of two independent cytotoxicity assays at an E:T ratio of 40:1. Bold-face type indicates significant cytotoxicity of targets when peptide-pulsed.

**Table 2** Cytotoxicity of peptide-specific CTL lines against cultured HLA-A2 and/or HLA-A3 breast cancer cells

| Target cell line (HLA-A type)[a] | CTL lines against GLQKASIFLK[c] | | | | | CTL lines against KASIFLKTRV | | | |
|---|---|---|---|---|---|---|---|---|---|
| Donor number[d]: HLA-A: | 5<br>2, 2 | 6<br>2, 3 | 7<br>2, 32 | 13<br>3, – | 14<br>3, 31 | 6<br>2, 3 | 15<br>2, – | 8<br>3, 11 | 16<br>3, 24 |
| BA ($2^+$, $3^-$)[b] | 64.6 ± 1.9[e] | 73.4 ± 2.1 | 52.8 ± 1.8 | 28.1 ± 1.5 | 42.7 ± 1.4 | 31.5 ± 1.3 | 23.2 ± 0.8 | 2.8 ± 0.4 | 10.2 ± 0.7 |
| BB ($2^+$, $3^-$)[b] | 43.9 ± 1.3 | 45.4 ± 1.6 | 40.6 ± 1.6 | 2.2 ± 0.3 | 4.4 ± 0.4 | 16.1 ± 1.2 | 18.6 ± 1.3 | 2.1 ± 0.3 | 5.7 ± 0.5 |
| BC ($2^+$,$3^-$)[b] | 67.7 ± 2.1 | 63.3 ± 1.9 | 47.7 ± 1.4 | 4.5 ± 0.3 | 6.1 ± 0.4 | 36.7 ± 1.5 | 19.3 ± 1.1 | 4.0 ± 0.3 | 6.9 ± 0.4 |
| BD ($2^+$, $3^-$)[b] | 20.2 ± 1.3 | 21.2 ± 1.5 | 18.2 ± 0.8 | 3.1 ± 0.4 | 4.7 ± 0.3 | 15.8 ± 1.1 | 13.5 ± 0.8 | 1.8 ± 0.2 | 5.3 ± 0.5 |
| BE ($2^+$, $3^-$)[b] | 49.5 ± 1.6 | 47.8 ± 1.3 | 45.3 ± 1.5 | 3.4 ± 0.2 | 3.2 ± 0.3 | 21.4 ± 0.9 | 18.3 ± 1.4 | 3.4 ± 0.2 | 4.0 ± 0.3 |
| BF ($2^-$, $3^+$)[b] | 6.1 ± 0.4 | 34.1 ± 1.4 | 8.5 ± 0.8 | 22.0 ± 1.1 | 33.4 ± 1.2 | 8.2 ± 0.6 | 4.9 ± 0.4 | 13.1 ± 1.0 | 10.8 ± 0.7 |
| BG ($2^-$, $3^+$)[b] | 4.8 ± 0.4 | 22.1 ± 1.0 | 5.8 ± 0.3 | 18.5 ± 1.0 | 24.8 ± 1.2 | 4.6 ± 0.3 | 2.2 ± 0.2 | 3.3 ± 0.4 | 4.1 ± 0.3 |
| BH ($2^-$, $3^-$)[b] | 2.6 ± 0.3 | 3.9 ± 0.3 | 4.6 ± 0.4 | 2.3 ± 0.2 | 2.9 ± 0.4 | 2.4 ± 0.4 | 2.7 ± 0.3 | 1.4 ± 0.1 | 4.8 ± 0.4 |
| BI ($2^-$, $3^-$)[b] | 7.7 ± 0.5 | 5.8 ± 0.3 | 6.2 ± 0.6 | 3.5 ± 0.4 | 7.0 ± 0.5 | 5.1 ± 0.6 | 4.5 ± 0.5 | 2.6 ± 0.3 | 6.6 ± 0.3 |
| BJ ($2^-$, $3^-$)[b] | 4.0 ± 0.2 | 3.3 ± 0.3 | 7.9 ± 0.6 | 4.2 ± 0.4 | 6.4 ± 0.4 | 3.9 ± 0.3 | 2.5 ± 0.2 | 2.0 ± 0.3 | 3.5 ± 0.3 |
| BK ($2^-$, $3^-$)[b] | 5.5 ± 0.5 | 6.3 ± 0.3 | 5.6 ± 0.4 | 2.6 ± 0.3 | 4.3 ± 0.4 | 5.5 ± 0.3 | 3.7 ± 0.2 | 3.4 ± 0.4 | 6.1 ± 0.4 |
| K562 | 5.7 ± 0.3 | 4.8 ± 0.4 | 8.1 ± 0.6 | 3.7 ± 0.3 | 6.8 ± 0.5 | 4.6 ± 0.4 | 4.1 ± 0.6 | 3.8 ± 0.3 | 5.3 ± 0.4 |
| 8392 | 4.3 ± 0.3 | 3.7 ± 0.4 | 3.2 ± 0.2 | 2.9 ± 0.4 | 4.9 ± 0.3 | 2.8 ± 0.4 | 2.4 ± 0.2 | 2.2 ± 0.4 | 3.4 ± 0.2 |

[a]BA is an established, long-term breast cancer cell line. BB to BK represent breast cancer primary cultures of less than four passages. K562 is a human erythroleukaemia cell line susceptible to LAK cells, and 8392 is an acute T lymphocytic leukaemia cell line resistant to LAK activity. [b]KASIFLK overexpression, as determined by immunohistochemical analysis using a human antibody to KASIFLK. [c]All CTL lines were tested for specific cytotoxicity after 4 weeks of stimulation with specific peptide. [d]Donor nos 5–8 are identical to the donors in Table 1. [e]Data are expressed as average per cent cytotoxicity and standard deviation of two independent cytotoxicity assays at an E:T ratio of 40:1.

N- and/or C-terminal of the antibody-recognizing heptameric sequence KASIFLK. GLQKASIFLK has anchor motifs for both HLA-A2 and HLA-A3. K at position 10, L at position 9 and L at position 2 are preferred anchor residues of HLA-A2. L at position 2 and K at position 10 are preferred anchor residues of HLA-A3. KASIFLKTRV also has anchor motifs for HLA-A2 and HLA-A3: A at position 2 and V at position 10 for HLA-A2, and A at position 2 and R at position 9 for HLA-A3. KASIFLKTR has a motif for HLA-A3 only: A at position 2 and R at position 9. PBMC from 41 normal healthy donors were stimulated with these peptides: 15 expressed HLA-A2, 10 expressed HLA-A3, four expressed HLA-A2 and HLA-A3 and 12 did not express HLA-A2 or HLA-A3 but did express other HLA-A motifs. These binding motifs were based on several published references (Celis et al, 1994*a*; Kawakami et al, 1994; Rammensee et al, 1995; Rivoltini et al, 1995; Sidney et al, 1996).

The cytotoxicity of each peptide-specific cell line was assessed using autologous BLC pulsed with the respective peptide. Stimulation by GLQKASIFLK, which has motifs for both HLA-A2 and HLA-A3, generated strong CTL from HLA-A2 donors and relatively weaker CTL from HLA-A3 donors. Cytotoxicity was greater than 20% for CTL generated from four of four HLA-A2, A3 donors, 11 of 15 HLA-A2 donors, and five of ten HLA-A3 donors. None of the CTL generated from 16 donors with other HLA types (HLA-$A2^-$, $A3^-$) showed specific cytotoxicity greater than 10%. These findings suggest that the recognition of GLQKASIFLK by CTL is restricted by both HLA-A2 and HLA-A3.

*© 1999 Cancer Research Campaign*

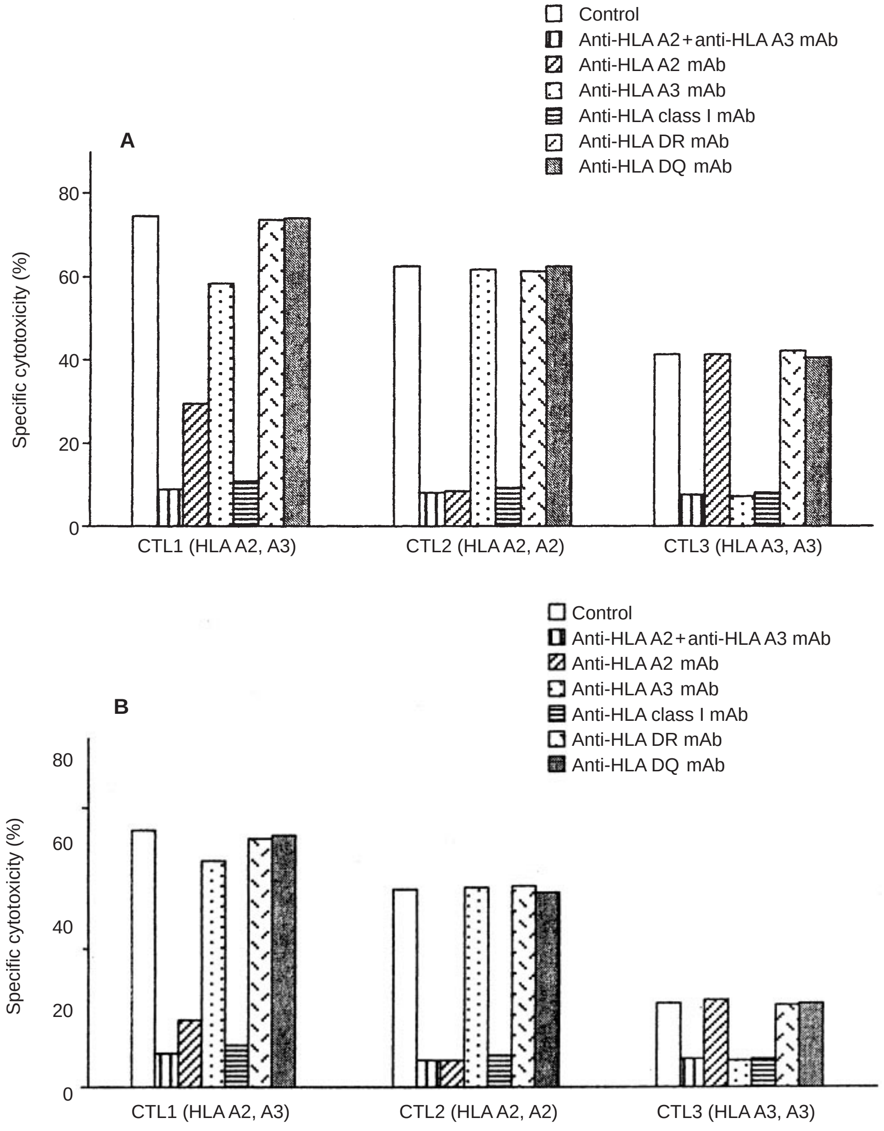

**Figure 1** Inhibition of cytotoxicity of GLQKASIFLK-specific CTL lines by anti-HLA mAb. PBMC from donor numbers 18 (HLA-A2, A2), 19 (HLA-A2, A3) and 20 (HLA-A3, A3) were stimulated with GLQKASIFLK for 16 weeks. Cytotoxicity against allogeneic HLA-A2, A3 breast cancer cell line JWCI-BA (**A**) and autologous BLC (**B**) was assessed at an E:T ratio of 40:1. Each anti-HLA mAb was used at a final concentration of 10 μg $ml^{-1}$. Cytotoxicity is shown as percent specific cytotoxicity. Background cytolysis of breast cancer cells expressing other HLA types and nonpulsed BLC was < 2.5%

CTL cultures that showed significant cytotoxicity were further stimulated with peptides and established into CTL lines. RBP-1 peptide KASIFLKTRV, which has a motif for both HLA-A2 and HLA-A3, was less effective than GLQKASIFLK in CTL generation. Cytotoxicity against peptide-pulsed BLC was greater than 20% for CTL generated from ten of 15 HLA-A2 donors but zero of ten HLA-A3 donors (data not shown). This suggests that KASIFLKTRV is presented primarily in the context of HLA-A2. The nonamer KASIFLKTR has a motif for HLA-A3 but showed only weak activity for eliciting peptide-specific CTL; only one CTL line (from an HLA-A3 donor) demonstrated cytotoxicity greater than 10%. Twelve representative CTL lines against the three peptides were consecutively generated from 12 different patients (Table 1).

The HLA restriction of CTL was then assessed using one established breast cancer cell line and ten breast cancer primary cell lines as targets. Five GLQKASIFLK-specific and four KASIFLKTRV-specific CTL lines, all derived from HLA-A2 or HLA-A3 PBMC, were tested for their cytotoxicity against seven HLA-A2 and/or HLA-A3 breast cancer cell lines, four HLA-A2$^{-}$, A3$^{-}$ breast cancer cell lines, K562 cell line (susceptible to LAK cells) and 8392 cell line (not susceptible to LAK cells). As shown in Table 2, the results further supported HLA-A2 and HLA-A3 restricted recognition of GLQKASIFLK and KASIFLKTRV, although HLA-A3 restricted recognition of the latter peptide was at a very low level. No significant lysis of breast cancer cell lines occurred when PBMC were cultured for 4 weeks without peptide or from HLA-A2$^{-}$, A3$^{-}$ PBMC stimulated with peptide (data not shown). The specific lysis of HLA-A2, A3 breast cancer cells by peptide-specific CTL was inhibited by mAb against HLA-A2, HLA-A3 or anti-HLA class I, but not by mAb against HLA-DQ or HLA-DR (Figure 1).

*© 1999 Cancer Research Campaign* 

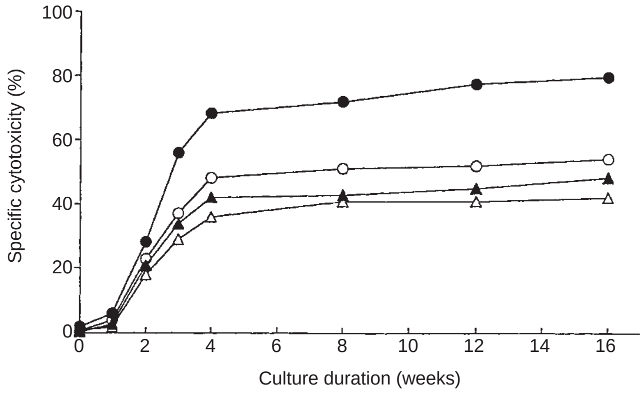

**Figure 2** Cytotoxicity of two representative peptide-stimulated CTL lines. PBMC from donor number 19 (HLA-A2, A3) were repetitively stimulated with GLQKASIFLK or KASIFLKTRV, and their toxicity against GLQKASIFLK-pulsed (▲) and KASIFLKTRV-pulsed (△) autologous BLC and against HLA-A2, A3 breast cancer cell line JWCI-BA (●, ○) was assessed at an E:T ratio of 40:1. Cytotoxicity against non-pulsed BLC was < 4.0% and subtracted from each peptide-pulsed BLC cytotoxicity

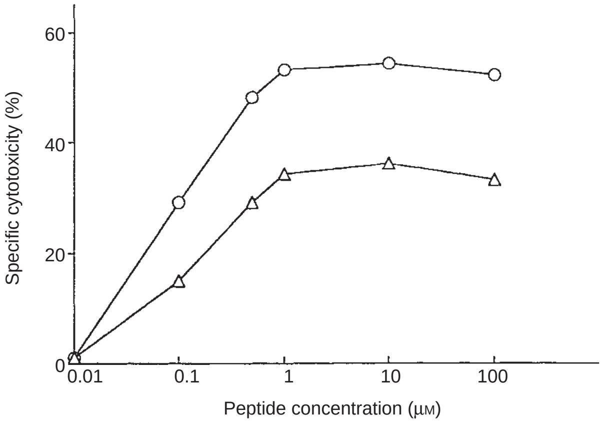

**Figure 3** Peptide dose-dependent lysis of target cells by peptide-specific CTL. Autologous BLC were pulsed with various concentrations of GLQKASIFLK (○) or KASIFLKTRV (△). Cytotoxicity assays were performed with respective peptide-specific CTL derived from donor number 21 (HLA-A2, A3) at an E:T ratio of 40:1. Background cytotoxicity of non-pulsed BLC was < 3.0%

**Table 3** Cytotoxicity of peptide-specific CTL lines against normal cells and a non-breast cancer cell line

| Target cell lines[a] | CTL lines against GLQKASIFLK[b] | | | CTL lines against KASIFLKTRV | | |
|---|---|---|---|---|---|---|
| Donor number: | 5 | 6 | 10 | 5 | 6 | 17 |
| HLA-A: | 2, 2 | 2, 3 | 3, 68 | 2, 2 | 2, 3 | 3, 29 |
| JWCI-BA breast cancer (HLA-A2, A3) | 72.4 ± 1.8[c] | 80.7 ± 1.7 | 46.3 ± 1.5 | 22.8 ± 1.3 | 38.3 ± 1.1 | 7.5 ± 0.6 |
| Fibroblast 1 (HLA-A1, A2) | 5.7 ± 0.4 | 6.2 ± 0.3 | 6.9 ± 0.4 | 3.7 ± 0.2 | 4.4 ± 0.4 | 2.2 ± 0.3 |
| Fibroblast 2 (HLA-A2, A3) | 6.1 ± 0.4 | 7.3 ± 0.4 | 5.4 ± 0.3 | 3.2 ± 0.3 | 3.7 ± 0.1 | 3.4 ± 0.2 |
| Fibroblast 3 (HLA-A2, A3) | 4.60 ± 0.3 | 5.6 ± 0.5 | 2.8 ± 0.1 | 2.9 ± 0.2 | 5.5 ± 0.3 | 2.9 ± 0.2 |
| Epidermal cell (HLA-A2, A3) | 2.6 ± 0.2 | 2.9 ± 0.3 | 2.0 ± 0.3 | 2.6 ± 0.3 | 1.7 ± 0.3 | 0.0 ± 0.2 |
| JWCI-MA melanoma (HLA-A2, A3) | 1.9 ± 0.2 | 2.2 ± 0.2 | 2.5 ± 0.3 | 2.9 ± 0.1 | 3.3 ± 0.3 | 3.2 ± 0.4 |

[a]Allogeneic cell lines of breast cancer, fibroblast, epidermal cell and melanoma had either HLA-A2 or HLA-A3 phenotype. JWCI-BA breast cancer cell line expresses the KASIFLK antigen. JWCI-MA melanoma cell line and normal cell lines were negative for this antigen. [b]All CTL lines were tested after 16 weeks of stimulation with peptide. Donor numbers correspond to those shown in Table 1. [c]Data are expressed as average per cent cytotoxicity and standard deviation of three independent cytotoxicity assays at an E:T ratio of 40:1.

### Kinetics of CTL maturation

Figure 2 shows the maturation pattern of representative CTL lines established by stimulation with each of the three RBP-1 peptides. Although a higher cytotoxicity was observed in cell lines stimulated with GLQKASIFLK than in cell lines stimulated with KASIFLKTRV, the pattern of stimulation was similar. After 4 weeks, cytotoxicity against breast cancer cells and peptide-pulsed BLC reached a plateau. The number of cells and specificity of the cell lines at week 4 decreased when peptide stimulation was interrupted, whereas CTL lines at week 16 were quite stable for both cell growth and specificity. The phenotyping of CTL lines against GLQKASIFLK or KASIFLKTRV at wk 16 revealed that these cells were predominantly $CD3^+$, $CD8^+$, $CD4^-$.

CTL lines stimulated for 16 wk with GLQKASIFLK and KASIFLKTRV were tested against autologous BLC pulsed with 0.01–100 μM of the corresponding peptide at an E:T ratio of 40:1 (Figure 3). Cytotoxicity increased in a dose-dependent manner until peptide concentration reached 1 μM.

### Assessment of cross-reactivity among three peptide-specific CTL

PBMC from an HLA-A2, A3 donor were stimulated for 16 weeks with each of the three RBP-1 peptides. The cytotoxic activity of the three ensuing CTL lines was assessed using an autologous BLC cell line pulsed with each peptide. As shown in Figure 4, GLQKASIFLK-specific and KASIFLKTRV-specific CTL lines had 51.2% and 38.1% specific cytotoxicity, respectively, against BLC pulsed with the respective stimulating peptide, but did not show significant cytotoxicity against BLC pulsed with the non-stimulating peptides. KASIFLKTR-specific CTL showed minimum cytotoxicity against KASIFLKTR-pulsed BLC (13.7%) and against the HLA-A2, A3 breast cancer cells (15.4%).

*© 1999 Cancer Research Campaign*

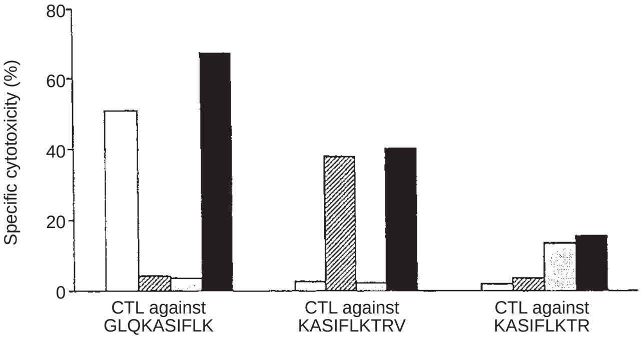

**Figure 4** Cross-reactivity of three peptide-specific CTL lines. PBMC from donor number 21 (HLA-A2, A3) were stimulated with each peptide for 16 weeks and tested for their cytotoxicity against autologous BLC pulsed with GLQKASIFLK (□), KASIFLKTRV (▨), KASIFLKTR (□), and JWCI-BA (■). Peptides were used at a final concentration of 10 μg $ml^{-1}$. Specific cytotoxicity is the mean of three independent assays performed at an E:T ratio of 40:1.

### Peptide-specific CTL activity against normal cells and melanoma cells

Autologous or allogeneic HLA-A2 or HLA-A3 matched normal cells (fibroblasts, epidermal cells and PBMC) and a melanoma cell line (HLA-A2, A3) were not recognized by GLQKASIFLK-specific or KASIFLKTRV-specific CTL (Table 3). Furthermore, while the cytotoxicity of both CTL lines was almost completely inhibited by cold target breast cancer cells at a cold:hot target cell ratio of 20:1, cytotoxicity was not affected by K562 cells, autologous normal fibroblasts or HLA-A2, A3 melanoma cells used as cold targets, even at the ratio of 80:1 (Figure 5).

### DISCUSSION

In this study we analysed CTL activity against specific peptide sequences of RBP-1. Previously, we demonstrated that an RBP-1 peptide sequence, KASIFLK, is recognized by human antibodies. KASIFLK peptide was detected by antibody staining in 12 of 15 (80%) breast cancer specimens, but not in 50 normal tissues (Cao et al, 1999). In the present study we demonstrate that the heptameric peptide KASIFLK is recognized by CTL. The inclusion of adjacent amino acids on the $NH_2$ or COOH terminal of KASIFLK provided the necessary length and HLA-A anchor motifs for HLA-restricted T-cell recognition. RBP-1 peptides GLQKASIFLK and KASIFLKTRV, both of which contain KASIFLK, stimulated peptide-specific CTL that recognized respective BLC peptide-pulsed target cells and breast cancer cell lines.

Presence of the peptide binding motifs does not always predict HLA-restricted CTL recognition. The peptide KASIFLKTR that contains HLA-A3 motifs, A at position 2 and R at position 9, did not induce an effective CTL response (Table 1). This may be explained by weak antigenicity and/or weak binding of the peptide to the HLA molecule. An effective peptide sequence for CTL induction depends on multiple factors. In our system, we can narrow the selection criteria by using a CTL peptide sequence built around an antigenic epitope recognized by a human antibody. The dual effector arms recognizing tumour-associated antigen peptides do not appear rare (Morioka et al, 1994; Takahashi et al, 1997*a*, 1997*b*), and such peptides may represent potential antigens for active specific immunotherapy.

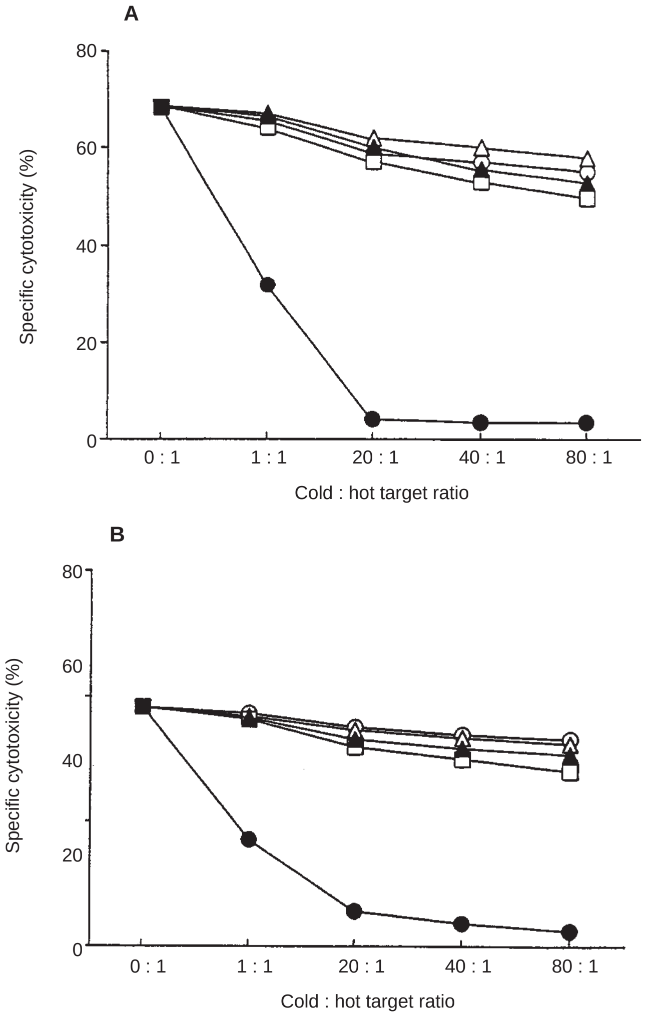

**Figure 5** Cold target inhibition of GLQKASIFLK-specific HLA-A2, A3 CTL against allogeneic HLA-A2, A3 breast cancer cell line JWCI-BA (**A**) and autologous GLQKASIFLK-pulsed BLC (**B**). The CTL line was derived from donor number 19 (HLA-A2, A3). Autologous PBMC (○), HLA-A2, A3 melanoma cells (△), autologous fibroblasts from normal dermal tissues (□), K562 cells (▲), and JWCI-BA breast cancer cell line (●) were used as cold targets. Cytotoxicity assays were performed at an E:T ratio of 40:1. Non-labelled cold targets were added at the indicated ratios to the hot targets

Immunohistochemical analysis has shown that the specific antibody recognizing the RBP-1 heptamer peptide sequence KASIFLK binds predominantly to breast cancer tissues and breast cancer cell lines (Cao et al, 1999). Although normal cells as well as other types of cancer cells express RBP-1 mRNA, the antibody failed to detect the epitope in these cells. We initially attributed this to the level of expression of the specific peptide sequence and/or to conformational or post-translational changes of the protein in breast cancer cells. Future studies will determine the RBP-1 protein expression levels and the post-translational modifications in normal and breast cancer cells. However, we have not excluded the possibility that other proteins expressing KASIFLK or cross-reactive peptides are responsible for the antibody staining of the proteins and/or CTL recognition of the peptides.

The overexpression of the RBP-1 peptide or cross-reactive peptides in breast cancer cells may play a major role in the CTL

*© 1999 Cancer Research Campaign*

antigenicity of breast cancer cells. The human antibody detects the core peptide antigen, KASIFLK, predominantly in the cytoplasm of breast cancer cells. Antigen presentation with HLA class I molecules requires cytosolic processing and transportation (Germain, 1994; Heemels and Pleogh, 1995). Therefore, CTL recognition of the peptides in breast cancer cells may be related to intracellular antigen processing that influences HLA presentation on the cell surface (Nijman et al, 1994).

We observed that RBP-1 peptide-specific CTL could be generated relatively easily by in vitro stimulation from HLA-A2 and HLA-A3 healthy donor PBL. This suggests that the peptide is quite antigenic. However, CTL killing was observed only against individual breast cancer cells, not against normal cells. A similar observation has been made for the tumour-associated antigens MAGE-1 and MAGE-3. MAGE-1 and MAGE-3 specific CTL can be generated from normal healthy donors as well as from the PBL of cancer patients; these CTL recognize tumour cells expressing moderate levels of the respective MAGE antigen (Traversari et al, 1992; Boon et al, 1994; Celis et al, 1994*b*). These observations indicate that the antigenic peptide sequence might be recognized only on tumour cells that overexpress the peptide.

RBP-1 can promote tumour growth by inhibiting the function of pRB. RBP-1 binds pRB at the binding motif (LXCXE) (Fattaey et al, 1993). Overexpression of RBP-1, as occurs in breast cancer, may promote active proliferation of tumour cells. CTL recognition of tumour cells expressing the RBP-1 peptide antigen would be important in developing active specific immunotherapies for prophylactic and therapeutic control of breast cancer. HLA-A2 and HLA-A3 molecules are found in high frequency in the general population and therefore offer a considerable advantage over a single HLA-A or HLA-B restricted peptide sequence (Hoon et al, 1998). Recently, several other tumour-associated proteins that are related to cell growth and cell cycle regulation, such as p53, HER2/NEU2 and p21 (RAS), have been shown to be antigenic (Nijman et al, 1994; Peace et al, 1994; Peoples et al, 1994; Gnjatic et al, 1995; Van Elsas et al, 1995). In future studies we will assess the in vivo immunogenicity of RBP-1 peptides by studying PBMC from breast cancer patients.

### ACKNOWLEDGEMENTS

This work was supported by a grant from the National Cancer Institute (CA12582) and by funding from the Ben B and Joyce E Eisenberg Foundation, Los Angeles, CA. The authors thank Lan Sze and Ingrid Ting for technical assistance.

### REFERENCES

Bookstein R, Shew JY, Chen PL, Scully P and Lee WH (1990) Suppression of tumorigenicity of human prostate carcinoma cells by replacing a mutated RB gene. *Science* **247**: 712–715

Boon T, Cerottini JC, Van den Eynde B, Van der Bruggen P and Van Pel A (1994) Tumor antigens recognized by T lymphocytes. *Annu Rev Immunol* **12**: 337–365

Cao J-N, Gao T, Giuliano AE and Irie RF (1999) Recognition of an epitope of a breast cancer antigen by human antibody. *Breast Cancer Res Treat* **53**: 279–290

Celis E, Fikes J, Wentworth P, Sidney J, Southwood S, Maewal A, Del Guercio M-F, Sette A and Livingston B (1994*a*) Identification of potential CTL epitopes of tumor-associated antigen MAGE-1 for five common HLA-A alleles. *Mol Immunol* **31**: 1423–1430

Celis E, Tsai V, Crimi C, De Mars R, Wentworth PA, Chesnut RW, Grey HM, Sette A and Serra HM (1994*b*) Induction of anti-tumor cytotoxic T lymphocytes in normal humans using primary cultures and synthetic peptide epitopes. *Proc Natl Acad Sci USA* **91**: 2105–2109

Defeo-Jones D, Huang PS, Jones RE, Haskell KM, Vuocolo GA, Hanobik MG, Huber HE and Oliff A (1991) Cloning of cDNA for cellular proteins that bind to the retinoblastoma gene product. *Nature* **352**: 251–254

Fattaey AR, Helin K, Dembski MS, Dyson N, Harlow E, Vuocolo GA, Hanobik MG, Haskell KM, Oliff A, Defeo-Jones D and Jones RE (1993) Characterization of the retinoblastoma binding proteins RBP1 and RBP2. *Oncogene* **8**: 3149–3156

Germain RN (1994) MHC-dependent antigen processing and peptide presentation: providing ligands for T-lymphocyte activation. *Cell* **76**: 287–299

Gnjatic S, Bressac-de Paillerets B, Guillet JG and Choppin J (1995) Mapping and ranking of potential cytotoxic T epitopes in the p53 protein: effect of mutations and polymorphism on peptide binding to purified and refolded HLA molecules. *Eur J Immunol* **25**: 1638–1642

Heemels MT and Ploegh H (1995) Generation, translocation, and presentation of MHC class I-restricted peptides. *Annu Rev Biochem* **64**: 463–491

Heskcth R (ed) (1997) *The Oncogene and Tumour Suppressor Gene Facts Book*, 2nd edn, pp 466–475. Academic Press: London

Hoon DSB, Okamoto T, Wang HJ, Elashoff R, Nizze JA, Foshag LJ, Gammon G and Morton DL (1998) Is the survival of melanoma patients receiving polyvalent melanoma cell vaccine linked to the human leukocyte antigen phenotype of patients? *J Clin Oncol* **16**: 1430–1437

Huang S, Lee WH and Lee EY (1991) A cellular protein that competes with SV40 T antigen for binding to the retinoblastoma gene product. *Nature* **350**: 160–162

Kaelin WG Jr, Pallas DC, DeCaprio JA, Kaye FJ and Livingston DM (1991) Identification of cellular proteins that can interact specifically with the T/E1A-binding region of the retinoblastoma gene product. *Cell* **64**: 521–532

Kaelin WG Jr, Krek W, Sellers WR, DeCaprio JA, Ajchenbaum F, Fuchs CS, Chittenden T, Li Y, Farnham PJ, Blanar MA, Livingston DM and Flemington EK (1992) Expression cloning of a cDNA encoding a retinoblastoma-binding protein with E2F-like properties. *Cell* **70**: 351–364

Kawakami Y, Eliyahu S, Aakaguchi K, Robbins PF, Rivoltini L, Yanelli JR, Appela E and Rosenberg SA (1994) Identification of the immunodominant peptides of the MART-1 human melanoma antigen recognized by the majority of HLA-A-2-restricted tumor infiltrating lymphocytes. *J Exp Med* **180**: 347–352

Lee EYP, To H, Shew JY, Bookstein R, Scully P and Lee WH (1988) Inactivation of the retinoblastoma susceptibility gene in human breast cancer. *Science* **241**: 218–221

Lee WH, Bookstein R, Hong F, Young LJ, Shew JY and Lee EYP (1987) Human retinoblastoma susceptibility gene: cloning, identification, and sequence. *Science* **235**: 1394–1399

Morioka N, Kikumoto Y, Hoon DSB, Morton DL and Irie RF (1994) A decapeptide (Gln-Asp-Leu-Thr-Met-Lys-Tyr-Gln-Ile-Phe) from human melanoma is recognized by CTL in melanoma patients. *J Immunol* **153**: 5650–5658

Nijman HW, Van der Burg SH, Vierboom MPM, Houbiers JGA, Kast WM and Melief CJM (1994) p53, a potential target for tumor-directed T cells. *Immunol Lett* **40**: 171–178

Otterson GA, Kratzke RA, Lin AY, Johnson PG and Kaye FJ (1993) Alternative splicing of the RBP1 gene clusters in an internal exon that encodes potential phosphorylation sites. *Oncogene* **8**: 949–957

Peace DJ, Smith JW, Chen W, You SG, Cosand WL, Blake J and Cheever MA (1994) Lysis of ras oncogen-transformed cells by specific cytotoxic T lymphocytes elicited by primary in vitro immunization with mutated ras peptide. *J Exp Med* **179**: 473–479

Peoples GE, Yoshino I, Douville CC, Andrews JVR, Goedegebuure PS and Eberlin TJ (1994) TCR V beta3$^+$ and V beta6$^+$ CTL recognize tumor-associated antigens related to HER2/neu expression in HLA-A2$^+$ ovarian cancers. *J Immunol* **152**: 4993–4999

Rammensee H-G, Friede T and Stevanovic S (1995) MHC ligands and peptide motifs: first listing. *Immunogenetics* **41**: 178–228

Rivoltini L, Kawakami Y, Sakaguchi K, Southwood S, Sette A, Robbins PF, Marincola FM, Salgaller ML, Yannelli JR, Appella E and Rosenberg SA (1995) Induction of tumor-reactive CTL from peripheral blood and tumor-infiltrating lymphocytes of melanoma patients by in vitro stimulation with an immunodominant peptide of the human melanoma antigen MART-1. *J Immunol* **154**: 2257–2265

Sidney J, Grey HM, Kubo RT and Sette A (1996) Practical, biochemical and evolutionary implications of the discovery of HLA class I supermotifs. *Immunol Today* **17**: 261–266

Takahashi T, Irie RF, Morton DL and Hoon DSB (1997*a*) Recognition of gp43 tumor-associated antigen peptide by both HLA-A2 restricted CTL lines and antibodies from melanoma patients. *Cell Immunol* **178**: 162–171

Takahashi T, Irie RF, Nishinaka Y and Hoon DSB (1997*b*) 707-AP peptide recognized by human antibody induces HLA-A2 restricted CTL killing of melanoma. *Clin Cancer Res* **3**: 1363–1370

 © 1999 Cancer Research Campaign

Traversari C, Van der Bruggen P, Luescher IF, Lurquin C, Chomez P, Val Pel A, De Plaen E, Amar-Costesec A and Boon T (1992) A nonapeptide encoded by human gene MAGE-1 is recognized on HLA-A1 by cytolytic T lymphocytes directed against tumor antigen MZ2-E. *J Exp Med* **176**: 1453–1457

Van Elsas A, Nijman HW, Van der Minne CE, Mourer JS, Kast WM, Melief CJM and Schrier PI (1995) Induction and characterization of cytotoxic T-lymphocytes recognizing a mutated p21ras peptide presented by HLA-A *0201. *Int J Cancer* **61**: 389–396

Wiman KG (1993) The retinoblastoma gene: role in cell cycle control and cell differentiation. *FASEB J* **7**: 841–845

*© 1999 Cancer Research Campaign*
